# Phosphorylation of GCN2 by mTOR confers adaptation to conditions of hyper-mTOR activation under stress

**DOI:** 10.1016/j.jbc.2024.107575

**Published:** 2024-07-14

**Authors:** Odai Darawshi, Olaya Yassin, Miri Shmuel, Ronald C. Wek, S. Jalil Mahdizadeh, Leif A. Eriksson, Maria Hatzoglou, Boaz Tirosh

**Affiliations:** 1 The School of Pharmacy, The Hebrew University of Jerusalem, Jerusalem, Israel; 2 Department of Biochemistry and Molecular Biology, Indiana University School of Medicine, Indianapolis, Indiana, USA; 3 Department of Chemistry and Molecular Biology, University of Gothenburg, Gothenburg, Sweden; 4 Department of Genetics and Genome Sciences, Case Western Reserve University, Cleveland, Ohio, USA; 5 Department of Biochemistry, Case Western Reserve University, Cleveland, Ohio, USA

**Keywords:** starvation, stress, translation regulation, protein docking, protein protein interactions

## Abstract

Adaptation to the shortage in free amino acids (AA) is mediated by 2 pathways, the integrated stress response (ISR) and the mechanistic target of rapamycin (mTOR). In response to reduced levels, primarily of leucine or arginine, mTOR in its complex 1 configuration (mTORC1) is suppressed leading to a decrease in translation initiation and elongation. The eIF2α kinase general control nonderepressible 2 (GCN2) is activated by uncharged tRNAs, leading to induction of the ISR in response to a broader range of AA shortage. ISR confers a reduced translation initiation, while promoting the selective synthesis of stress proteins, such as ATF4. To efficiently adapt to AA starvation, the 2 pathways are cross-regulated at multiple levels. Here we identified a new mechanism of ISR/mTORC1 crosstalk that optimizes survival under AA starvation, when mTORC1 is forced to remain active. mTORC1 activation during acute AA shortage, augmented ATF4 expression in a GCN2-dependent manner. Under these conditions, enhanced GCN2 activity was not dependent on tRNA sensing, inferring a different activation mechanism. We identified a labile physical interaction between GCN2 and mTOR that results in a phosphorylation of GCN2 on serine 230 by mTOR, which promotes GCN2 activity. When examined under prolonged AA starvation, GCN2 phosphorylation by mTOR promoted survival. Our data unveils an adaptive mechanism to AA starvation, when mTORC1 evades inhibition.

Eukaryotic cells adapt their metabolism to nutrient availability to ensure survival and function. Amino acids (AA) are essential for mammalian cells and are regulated through various processes including transport, synthesis, and degradation. Cells adjust protein synthesis based on AA levels, which are regulated by two major kinases: General control nonderepressable 2 (GCN2) and mechanistic target of rapamycin (mTOR) (1).

When AA levels are in shortage, GCN2 is activated by uncharged tRNA molecules. The tRNAs bind GCN2 through the histidyl-tRNA-like domain, triggering an ordered mechanism of activation involving a self-phosphorylation at Thr-899, situated in the activation loop of the GCN2 protein kinase. Activated GCN2 then catalyzes the phosphorylation of the α subunit of eukaryotic initiation factor 2 (eIF2) at serine 51, triggering translational control. This phosphorylation is shared with three additional eIF2α kinases: protein kinase R (PKR), protein kinase RNA-Like ER kinase (PERK), and heme-regulated eIF2α kinase (HRI) each phosphorylates eIF2α in response to different stress conditions. Thus, the phosphorylation of eIF2α was coined the integrated stress response (ISR) (2). Phosphorylated eIF2α confers a reduction in the guanine nucleotide exchange factor activity of eIF2B, which leads to a reduction in the levels of the ternary complex necessary for translation initiation. ISR is therefore associated with a global reduction in protein synthesis. At the same time, by virtue of proximity between upstream open reading frames (uORFs) and main ORFs, an advantage in translation is imparted for certain mRNAs, including that encoding ATF4, a potent transcription factor, which is considered as the *bona fide* marker of the ISR (3).

mTOR is a serine/threonine protein kinase that belongs to the PI3K-related protein kinases family. In mammals, it constitutes the catalytic subunit of two distinct complexes known as mTOR complex 1 (mTORC1) and mTORC2 (4, 5). Of these complexes, mTORC1 is a major metabolic regulator, which promotes the biosynthesis of protein, lipids, and nucleic acids by phosphorylating key substrates, including 4E-BP1 and S6K1. mTORC1 also funnels multiple inputs on cellular metabolism, including levels of AAs. In contrast to GCN2, which responds to a scarcity in a wide range of AAs, mTORC1 largely responds to levels of leucine and arginine (6, 7). The pathway by which levels of these specific AAs are conveyed to mTORC1 activity is controlled primarily by cytoplasmic sensors that interact with upstream regulatory complexes of mTORC1. A major regulatory complex is GATOR1, composed of Nprl2, Nprl3 and DEPDC5 (8). Since GATOR1 inhibits mTORC1, the deletion of its subunits renders mTORC1 hyperactive and insensitive to a reduction in leucine or arginine levels (9).

Strong chronic activation of ISR or severe inhibition of mTORC1 can be toxic in most cell types. Hence, as a mechanism of adaptation, these pathways are intrinsically regulated. ATF4 plays a central role in this adaptation by preventing excessive ISR activation. A major mechanism is by directly activating the expression of GADD34, which facilitates the timely dephosphorylation of eIF2α and terminates the ISR (10). In addition, ATF4 elevates the expression of AA transporters, such as Slc7a5 and Slc7a11 (11). Simultaneously, ATF4 inhibits mTORC1 activity by promoting the expression of negative regulators, such as Sestrin2 and Redd1 (12). This facilitates autophagy to further elevate the levels of cellular-free AAs. Noteworthy, the expression of ATF4 in mammalian cells is not solely dependent on the ISR, and can be induced, for instance, by insulin in an ISR-independent, mTORC1-dependent manner (13).

The cross-regulation between ISR and mTORC1 also involves ATF4-independent mechanisms. In yeast, which express TOR1 and GCN4 as orthologs of mTOR and ATF4, respectively, inhibition of TORC1 by rapamycin elevated the activity of GCN2 in a phosphorylation-regulated manner on residue 577 (14). Consistent with these findings, activation of GCN2 in the fission yeast suppresses TORC1 activity (15). In *Saccharomyces cerevisiae*, a mechanism that involves a direct phosphorylation of the TORC1 complex by GCN2 was suggested (16). It should be noted that key differences between fission and budding yeast and mammals exist, including the absence of these phosphorylations in mammalian cells (17).

The details of the cross-regulation between mTORC1 and ISR in mammalian cells are less understood. Deletion of GCN2 in cells and in mice results in strong activation of mTORC1 under reduced AA levels, as imparted for instance by asparaginase (18), by unknown mechanisms. A pharmacological screen identified inhibitors of mTORC1 that indirectly inhibit GCN2 through translation inhibition (19). While a direct interaction between GCN2 and mTOR in mammalian cells has not been demonstrated, recent findings show that GCN2 promotes ubiquitination of mTOR in response to amino acid starvation, impeding its interactions with substrates 4E-BP1 and S6K1 (20). Thus, similar to yeast, in mammalian cells, GCN2-dependent ISR and mTORC1 respond to stress in opposite manners. GCN2 is activated while mTORC1 is inhibited (21), and both responses cooperate to inhibit protein synthesis and assist in adaptation, primarily through ATF4-mediated activities (12). However, in some cellular circumstances, the coordination between the two pathways may go awry, through mutations or activation of mTORC1 by AA-independent conditions.

We recently showed that when high mTORC1 activity is enforced by deletion of the tuberous sclerosis complex subunit TSC2, cells readily develop mitochondrial stress, which compromises cell viability. Under these conditions, the eIF2α kinase HRI is activated and provides protection (22). Here we addressed the cellular response to AA starvation under conditions of enforced high mTORC1 activity, achieved genetically by deletion of the GATOR1 subunit NPRL2, or TSC2. We reasoned that similar to mitochondrial stress and HRI activation, under AA starvation conditions, GCN2 should provide cytoprotection. We confirmed an enhanced activation of GCN2 when mTORC1 activity is enforced upon AA starvation, which strongly promoted the expression of ATF4. Biochemical analyses supported by molecular modeling identified a direct interaction between mTOR and GCN2. Our data show that GCN2 is a substrate of mTOR undergoing novel phosphorylation that is needed for adaptation to conditions of hyper-mTORC1 activity under AA starvation.

## Results

### mTORC1 and GCN2 synergize to promote ATF4 activity under starvation conditions

**Figure 1:**
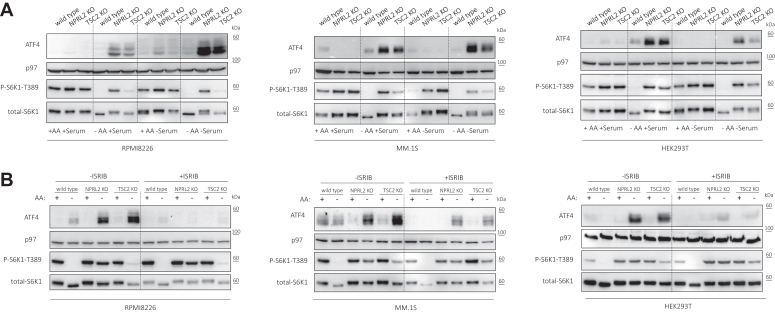
**Genetic activation of mTORC1 enhances the ISR during acute AA starvation.***A*, wild-type, NPRL2 KO, and TSC2 KO of RPMI8226, MM.1S, and HEK293T cells were cultured in AA replete, or AA deplete conditions with or without dialyzed serum for 1 h, as indicated. *B*, wild-type, NPRL2 KO, and TSC2 KO cells of the indicated cell lines were cultured in AA replete, or AA deplete conditions and either treated with ISRIB [1 μM] or left untreated as a control. Shown are representative immunoblots of the indicated proteins, p97 immunoblots are shown as a loading control.

**Figure 2:**
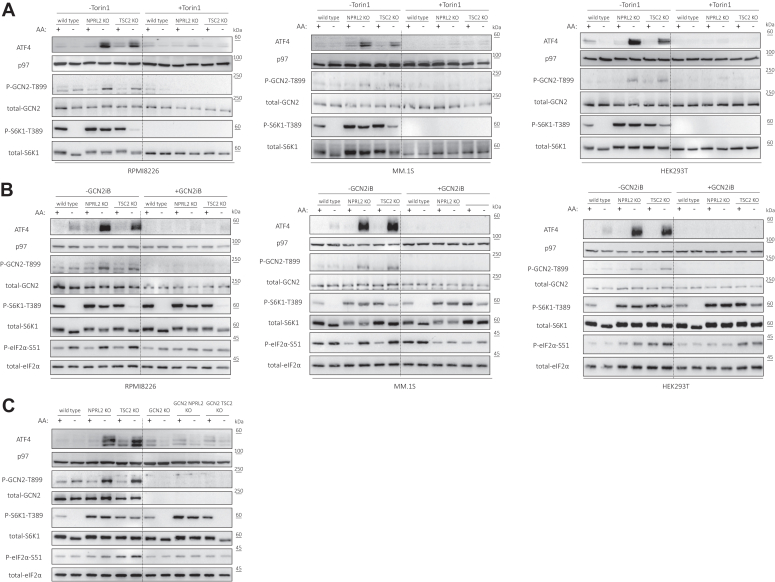
**mTORC1 activation induces the ISR in a GCN2-dependent manner upon acute AA starvation.***A*, Wild-type, NPRL2 KO, and TSC2 KO cells of the indicated cell lines were cultured in AA replete, or AA deplete conditions and either treated with Torin1 [1 μM] or *left* untreated as a control. *B*, Wild-type, NPRL2 KO, and TSC2 KO cells of the indicated cell lines were cultured in AA replete, or AA deplete conditions and either treated with GCN2iB [1 μM] or left untreated as a control. *C*, Wild-type, NPRL2 KO, and TSC2 KO HEK293T cells were subjected to genetic deletion of GCN2 and cultured in AA replete, or AA deplete conditions for 1 h. Shown are representative immunoblots of the indicated proteins, p97 immunoblots are shown as a loading control.

AA starvation induces the phosphorylation of eIF2α and ATF4 expression in a GCN2-dependent manner. However, this regulation typically requires 2 to 3 h (23, 24). Induction in the mRNA level of ATF4 requires longer durations of treatment (25). To examine the contribution of mTORC1 to ISR activity, we focused on measuring the expression levels of ATF4 under acute starvation conditions. We limited the starvation of either AA, dialyzed serum, or both to 1 h using three different cell lines and their genetic knockout (KO) models of NPRL2 and TSC2 (Figs. 1*A* and S1). As expected, this starvation duration was sufficient to abolish mTORC1 signaling in wild-type (WT) cells but not in cells that hyperactivate mTORC1, as measured by immunoblotting of the phosphorylation of its downstream target S6K1 at threonine 389 (P-S6K1-T389) (Fig. 1*A*). This effect was consistently observed across different cell lines during AA starvation but not serum starvation. ATF4 is subject to post-translational modifications, which may result in multiple bands on SDS-PAGE. Additionally, its basal levels can fluctuate under different uncontrolled conditions. During AA starvation, ATF4 expression was markedly elevated in cells with hyperactive mTORC1 compared to their WT counterparts (Fig. 1*A*). This effect was also observed in mouse cell lines, suggesting its generality (Fig. S2). Depleting serum further elevated ATF4 levels in RPMI8226 and MM.1S, but not in HEK293T cells (Fig. 1*A*), suggesting a potential correlation between stress severity and response. To determine whether ATF4 expression was mediated by the ISR, we combined the starvation with ISRIB, an eIF2B activator that attenuates the ISR (26, 27). We found that ATF4 induction was markedly reversed by ISRIB treatment without affecting mTORC1 or GCN2 activities (Figs. 1*B* and S4). We then examined whether ATF4 expression is dependent on GCN2 or mTOR activities. Inclusion of Torin1, which blocks mTORC1/2, obliterated ATF4 induction, indicating dependency on mTOR activity (Fig. 2*A*). GCN2 was markedly more activated in the cells that hyperactivate mTORC1 compared to their WT counterparts upon AA starvation, as measured by immunoblotting of the phosphorylation of GCN2 at threonine 899 (P-GCN2-T899). This activation was strongly reversed by Torin1 treatment, suggesting that mTOR activity influences GCN2 activation (Fig. 2*A*). To better understand the role of GCN2, we used the GCN2 inhibitor, A92. A92 was primarily used at a concentration of 10 μM, which is the highest concentration that does not activate PERK, the endoplasmic reticulum stress-coupled eIF2α kinase (28). While it blocks both GCN2 and ATF4 induction upon AA starvation, we found that it also has an inhibitory effect on mTORC1 (Fig. S3*A*), even at lower concentrations (Fig. S3*B*). To achieve more selective inhibition, we used GCN2iB. GCN2iB treatment abolished GCN2 and ATF4 induction and the increase in P-eIF2α-S51 upon AA starvation, however, it resulted in a mild activation of mTORC1 (Figs. 2*B* and S3*C*). Similarly, genetic deletion of GCN2 from both WT and mTORC1-hyperactie HEK293T cells had a similar effect (Fig. 2C). Based on these findings, we concluded that activation of mTORC1 augments a GCN2-dependent ISR upon acute AA starvation. We decided to focus on how genetic overactivity of mTORC1 affects the GCN2 arm of ISR.

### Activation of GCN2 by mTORC1 does not require tRNA sensing

**Figure 3:**
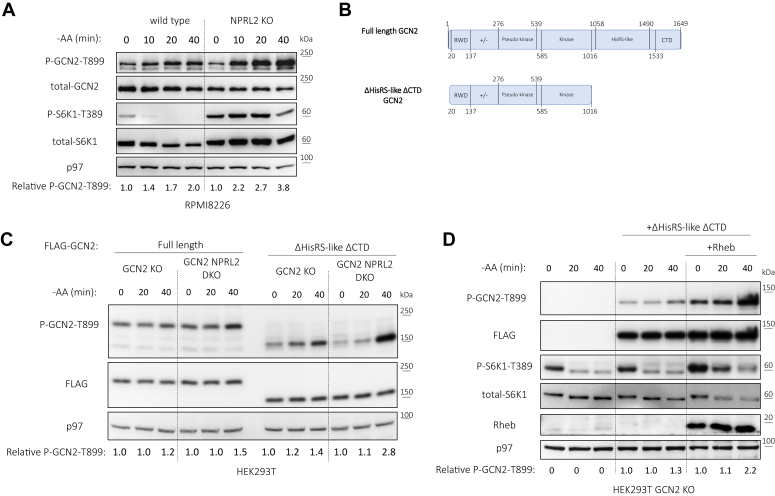
**mTORC1 overactivity promotes GCN2 autophosphorylation regardless of its binding ability to uncharged tRNAs or ribosomal proteins.***A*, Wild-type NPRL2 KO RPMI8226 cells were cultured in AA deplete medium for the indicated time points. GCN2 autophosphorylation was assessed by immunoblotting against P-GCN2-T899 and total-GCN2. mTORC1 activity was assessed by immunoblotting against P-S6K1-T389 and total-S6K1. *B*, human GCN2 protein domain organization scheme of full length and truncated used in (*C* and *D*). *C*, the indicated constructs of GCN2 were transfected into the indicated cells and their autophosphorylation on T899 was assessed as described in (*A*). *D*, HEK293T GCN2 KO cells were transfected with the indicated proteins. GCN2 and mTORC1 activities were assessed as described in (*A*). Shown are representative immunoblots and relative quantification of P-T899-GCN2 (to time 0).

We found that enforced activation of mTORC1 increases GCN2 autophosphorylation upon AA starvation (Figs. 2 and 3*A*). Canonical activation of GCN2 involves a direct binding to uncharged tRNAs and/or stalled ribosomes, both require the histidyl-tRNA synthetase-related domain (HisRS-like) and C-terminal domain (CTD) for binding and activation (29, 30). We expressed a truncated GCN2 that lacks both HisRS and CTD domains in HEK293 cells that are either knocked out for GCN2 or for GCN2 and NPRL2 (Fig. 3, *B* and *C*). We found that when mTORC1 activity is enhanced, autophosphorylated GCN2 levels are increased regardless of HisRS and CTD domains, consistent with their dispensable for activation by GCN2iB (24).

Rheb is a G protein necessary for mTORC1 activity and its overexpression is sufficient to induce mTORC1 activity (31). When this approach was applied to HEK293T cells that lack GCN2, the truncated GCN2 underwent hyperactivation (Fig. 3*D*). However, overexpressing Rheb in HEK293T WT cells did not enhance the autophosphorylation of endogenous GCN2 upon AA starvation, and this effect was not altered by Torin1 treatment (Fig. S5). We conclude that the mechanism by which mTORC1 promotes GCN2 may not involve uncharged tRNA sensing.

### mTOR and GCN2 directly interact

**Figure 4:**
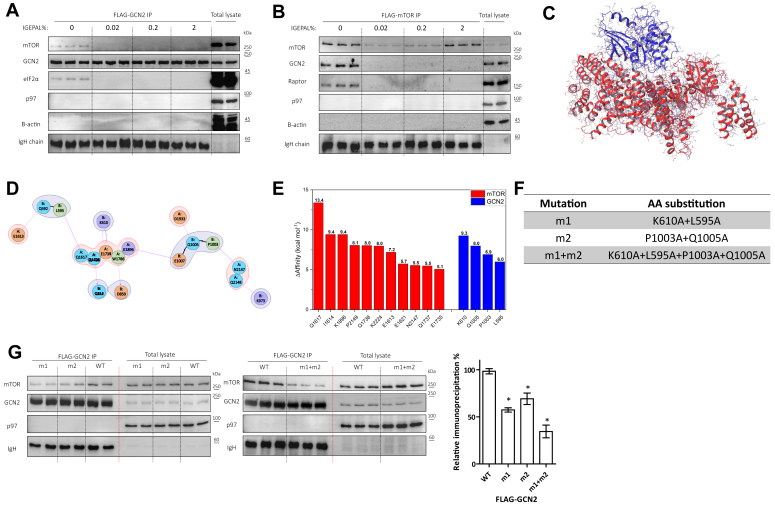
**Direct interaction between mTOR and GCN2.***A* and *B*, protein complexes were extracted in detergent-free lysis buffer following transfection of FLAG-GCN2 (*A*) or FLAG-mTOR (*B*) into HEK293T cells. Lysates were incubated with flag beads in different concentrations of the detergent IGEPAL as indicated. Shown are representative immunoblots of both flag-immunoprecipitated proteins (n = 3 for each condition, technical replicates) and their total lysates (n = 2, technical replicates) of Flag-GCN2 (*A*) or Flag-mTOR (*B*) transfected cells. *C*, *in silico* docking of the most populated cluster of tranucated-GCN2 (*blue*, 7QWK) with truncated-mTOR (*red*, 4JSV). The interacting residues were computationally identified and shown in (*D*). *E*, residues in the interface of GCN2 and mTOR were computationally mutated to alanine and change in affinity (Δaffinity) was calculated for each mutation. Among these, 3 mutants of GCN2 were experimentally prepared (*F*). *G*, the indicated mutants were transfected into HEK293T GCN2 KO cells and immunoprecipitated. Shown are representative immunoblots of both FLAG-immunoprecipitated proteins and their total lysates. Immunoglobulin heavy chain (IgH) immunoblot was used to evaluate loading quantity of FLAG-beads. The column graph represents the relative immunoprecipitation between mTOR and the different variants of FLAG-GCN2. Columns represent the percentage average ± SD.

In *S. cerevisiae*, Gcn2 transiently interacts with TORC1 upon AA starvation and phosphorylates Kog1, the yeast ortholog of the mammalian raptor (16). The exact configuration of the interaction was not solved. Since both mTOR and GCN2 are evolutionary conserved, we hypothesize that the kinases may also exert interactions in mammalian cells. To test this possibility, we applied hypotonic conditions to lyse the cells in the absence of detergent. HEK293T cells were transfected with FLAG-tagged GCN2 or FLAG-tagged mTOR. FLAG immunoprecipitations showed that GCN2 interacts with mTOR and *vice versa*, in the absence of the non-ionic detergent IGEPAL. Of interest, addition of very low concentrations of IGEPAL were sufficient to disrupt the interaction (Figs. 4, *A* and *B* and S6*A*). This interaction was also observed in other cell line models (Fig. S6*H*), suggesting its generality.

To begin addressing whether the interaction between GCN2 and mTOR is direct, we performed dynamic simulations between the 2 proteins using the publicly available crystal structures of their truncated forms (Fig. S6, *B*–*F*). These analyses identified a complementarity between the interphases of the 2 proteins (Fig. 4*C*). 2D projection and computerized alanine scanning identified four residues in GCN2 protein kinase domain that may contribute to the interaction: L595, K610, P1003, Q1005 (Fig. 4, *D* and *E*). To assess their contribution for the interaction, we generated 2 mutants, each containing 2 replacements to alanine (Fig. 4*F*). Immunoprecipitation analyses showed that in either one, m1 (K610A + L595A) or m2 (P1003A + Q1005A), the interaction of GCN2 was reduced. When the 4 mutations were combined (m1+m2), the interaction with mTOR was compromised to a larger extent (Fig. 4*G*). These results provide further support for a direct interaction between GCN2 and mTOR. Analyzing the activity of GCN2, by virtue of measuring its autophosphorylation level, we found that the m1 mutant was inactive (Fig. S6*G*). Overall, these findings provide evidence of direct, labile, interactions between mTOR and GCN2 in mammalian cells. Since only m1+m2 mutant interacted less with mTOR, we were unable to assess the effect of interaction on GCN2 activity under AA starvation conditions.

### mTOR phosphorylates GCN2 at S230

**Figure 5:**
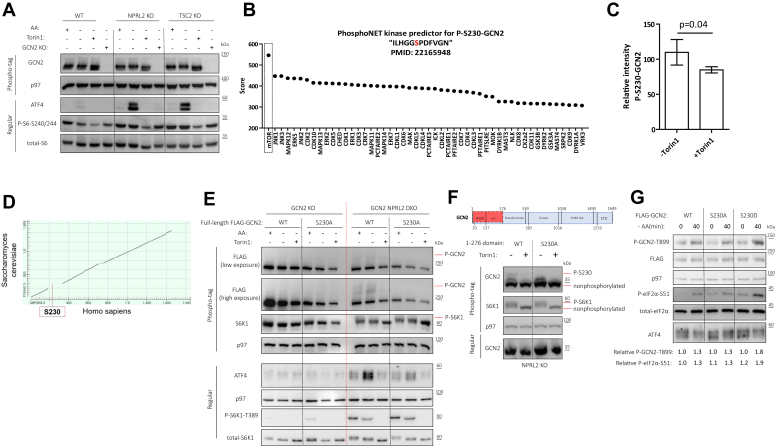
**mTOR phosphorylate GCN2atS230.***A*, the indicated HEK293T cell variants were cultured in AA replete, or AA deplete conditions and treated with Torin1 [1 μM] as indicated. Phospho-tag gel (12.5%) was used to evaluate the overall phosphorylation of GCN2; retarded migration of band reflects more phosphorylated protein and *vice versa*. *B*, kinase prediction ranking of the S230-GCN2 motif. *C*, the relative intensity of the phosphorylated S230 peptide was determined through mass spectrometry of FLAG-GCN2 immunoprecipitated from HEK293T NPRL2 KO cells that were starved from AA for 1 h and either treated with Torin1 [1 μM] or *left* untreated as a control. Each column represents 3 biological replicates ± SD. The *p*-value was calculated using a one-tailed Type II Student's *t* test. *D*, GCN2 protein alignment of *Homo sapiens* (human) and *Saccharomyces cerevisiae* (yeast), lines represent conversative sequence while gaps represent distinct sequences. S230 motif is emphasized as distinct to human. *E*, WT and S230A mutant of full-length FLAG-GCN2 were transfected into HEK293T GCN2 KO and GCN2 NPRL2 DKO cells. These cells were cultured in AA replete, or AA deplete conditions and treated with Torin1 [1 μM] as indicated. Shown are phospho-tag (7.5%) and regular representative immunoblots. *F*, the indicated domain of GCN2 was cloned and subjected to S230A mutation. Shown are phospho-tag (12.5%) and regular representative immunoblots of HEK293T NPRL2 KO cells expressing either WT or S230A of this domain and treated with Torin1 [1 μM] for 1 h or *left* untreated as a control. *G*, HEK293T GCN2 KO cells were transfected with the indicated full-length FLAG-GCN2 and cultured in the indicated conditions. Shown are representative immunoblots and relative quantification of P-GCN2-T899 and P-eIF2α-S51.

We proposed that the mechanism by which mTORC1 promotes GCN2 activity is a consequence of the interaction and may involve the phosphorylation of GCN2 by mTORC1. To determine the phosphorylation status of GCN2, we performed an analysis using phospho-tag (p-tag) SDS-PAGE, a technique that causes phosphorylated proteins to migrate more slowly, resulting in an upshifted band. Our results indicated that GCN2 exhibits a mild retardation of electromobility in cells subjected to AA starvation, suggesting increased phosphorylation. This upshift is observed specifically in cells that hyperactivate mTORC1, and it is sensitive to Torin1, indicating that the phosphorylation event is dependent on mTOR activity (Fig. 5*A*). PhosphoNET and PhosphoSite analyses predicted that the S230 residue fits best the mTOR motif (Figs. 5*B* and S7*A*). To examine this possibility, we expressed FLAG-tagged GCN2 in NPRL2 KO HEK293T cells. Following immunoprecipitation from cells treated or not with Torin1, we performed a phospho-protein mass spectrometry. A peptide that contains serine 230 was detected (Fig. S7*B*). The levels of this peptides decreased in cells treated with Torin1, compared to control treatment (Fig. 5*C*). It should be noted that S230 is located in the evolutionary non-conserved linker domain, which is absent from the *S. cerevisiae* TOR protein (Fig. 5*D*). Serine 230 is located in an unstructured flexible region for which there is no crystal structure (Fig. S7*C*). To analyze the phosphorylation at site S230 in the absence of a P-GCN2-S230 antibody, we employed p-tag SDS PAGE. We created a phosphorylation-deficient mutant, S230A, to serve as a negative control. Both WT GCN2 and the S230A mutant were expressed in GCN2 KO and NPRL2-GCN2 DKO HEK293T cells. Following AA starvation and treatment with Torin1, we observed an upshifted band corresponding to phosphorylated GCN2 in NPRL2-GCN2 DKO cells expressing the WT GCN2, but not in cells expressing the S230A mutant. This upshift was sensitive to Torin1 treatment, indicating that the phosphorylation of GCN2 at S230 is dependent on mTOR. Moreover, ATF4 expression was induced more in NPRL2 KO cells expressing WT GCN2 compared to those expressing the S230A mutant, but this difference was not observed in WT cells (Fig. 5*E*). In addition, we truncated GCN2 for improved resolution on SDS-PAGE, focusing on the first 276 AAs. When cloned and expressed in HEK293T cells, truncated WT GCN2 yielded 2 hyperphosphorylated bands. When the S230A mutant was examined, the top band was eliminated, suggesting that this band reflects a phosphorylation on S230. A treatment with Torin1, which blocks mTOR, decreased both phosphorylations, suggesting that at least 2 mTOR-dependent phosphorylation sites exist in GCN2, one is at position 230 (Fig. 5*F*). Furthermore, *in vitro* kinase assays, in which immunoprecipitated 1 to 276 domain of WT and S230A FLAG-GCN2 were incubated with immunoprecipitated mTOR and Rheb, exhibited ATP-dependent phosphorylation bands for WT GCN2 that were not present in the S230A mutant (Fig. S7*D*). These results support the model that mTOR directly phosphorylates GCN2 on serine 230.

To assess the impact of phosphorylation at serine 230 on GCN2 activity, we expressed FLAG-tagged, full-length WT GCN2, the S230A mutant, and a phosphomimetic S230D mutant in GCN2 KO HEK293T cells. During AA starvation, the S230D mutant exhibited increased activation compared to the WT and S230A, as evidenced by enhanced autophosphorylation. Additionally, the S230D mutant was more effective in phosphorylating eIF2α compared to the WT and S230A. However, there was no significant change in ATF4 expression within 40 min of AA starvation (Fig. 5*G*). This suggests that activation of mTORC1 in the presence of AA starvation is not fully recapitulated by the phosphorylation of GCN2 at S230, and additional mTOR-dependent events are required for the induction in ATF4 expression. Regardless the exact mechanisms, these observations suggest that mTORC1-mediated phosphorylation of GCN2 on S230 synergizes with AA starvation to promote GCN2 activity and is an important element in the events by which mTORC1 fortifies the ISR under AA starvation conditions.

### An S230D phosphomimetic GCN2 compromises cell growth under optimal culturing conditions but promotes survival upon AA starvation

**Figure 6:**
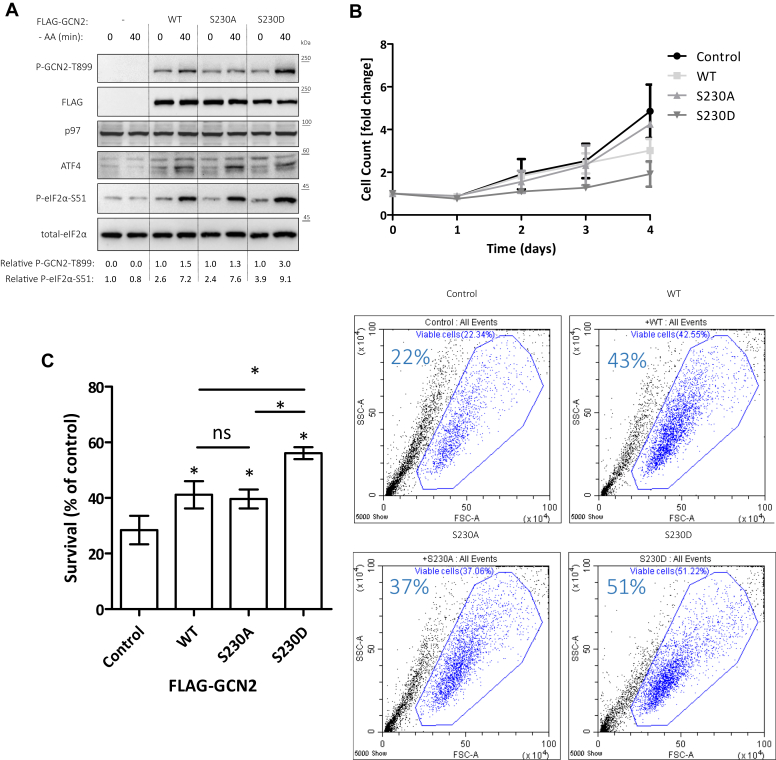
**PhosphorylationatS230 mounts GCN2 activity and promotes the survival of cells in AA starved conditions.***A*, MIA PaCa-2 GCN2 KO cells were transduced with the indicated full-length FLAG-GCN2 and cultured in the indicated conditions. Shown are representative immunoblots and relative quantification of P-GCN2-T899 and P-eIF2α-S51. *B*, cell counting following transduction of MIA PaCa-2 GCN2 KO cells with the indicated full-length FLAG-GCN2. (*C*) Cells described in (*A* and *B*) were starved from AA for 24 h. Column graph represents the average viability of 3 independent experiments ± SD, ∗*p* < 0.05 of unpaired two-tailed student’s *t* test. ns, not significant. Flow cytometry dot plots represent the viable cells percentage following the starvation.

We have previously shown that multiple myeloma cells, deficient for TSC2, gain sensitivity to proteasome inhibitors and hypoxia (22). We subjected these cells to AA starvation. For both cell types, RPMI8226 and MM.1S, and in both mTORC1-induced variants, NPRL2 and TSC2 knockouts, hypersensitivity to AA starvation was observed (Fig. S8*A*). We decided to test this also in pancreatic cancer cells. Pancreatic cancer is an incurable disease, characterized by a developed parenchyma that is poorly vascularized (32, 33). It was suggested that pancreatic cancer progress despite conditions of nutrient starvation. We decided to examine the importance of mTORC1/GCN2 interaction for growth and survival in MIA PaCa-2 pancreatic cancer cells. We generated NPRL2 and TSC2 KO variants of MIA Paca-2 cells (Fig. S8*B*). Hypersensitivity to AA starvation was observed, similar to the myeloma cells (Fig. S8*C*). We then generated GCN2 KO in MIA PaCa-2, followed by reconstitution with FLAG-GCN2 that is either WT, S230A or S230D. Similar to HEK293T cells, S230D mutant showed higher activity than WT or S230A, however, without a significant difference in ATF4 expression (Fig. 6*A*), further demonstrating that high mTORC1 supports ATF4 expression by the cooperation of GCN2-dependent and -independent events. It has been shown that constitutive ISR compromises cell growth, and tumors devise strategies to eliminate the ISR (34). When examined under normal culturing conditions, cell proliferation was compromised in S230D expressing MIA PaCa-2 cells (Fig. 6*B*). However, when stress was applied by virtue of AA depletion for 24 h, S230D expressing cells endured it better than WT or S230A expressing cells (Fig. 6*C*). Our data implicate the phosphorylation on S230 in GCN2 as may provide a safety adaptive mechanism for conditions of AA starvation, when mTORC1 activity failed to be suppressed.

## Discussion

mTOR activity is connected to cell physiology at multiple levels. mTORC1 is downstream to growth factor signaling through the tyrosine kinase receptors (RTK), PI3 kinases (PI3K), and the kinase Akt. The RTK-PI3K-Akt-mTOR constitutes a major oncogenic pathway. While mutations in mTOR are rare in cancer, this axis is often activated genetically upstream to mTOR to promote uncontrolled growth (35). The ISR effects and its role in cell fitness are cell type-specific. Under various toxic stress conditions, the ISR is typically cytoprotective (21). In contrast to standard tissue culture media, the availability of free AA *in vivo* is more limited and cells utilize both mTORC1 and GCN2 to regulate their growth under fluctuated nutritional availability, with direct implications for cancer (21, 32). Thus, although mTORC1 and GCN2 evolved to adjust metabolism to a limited supply of AAs, the responses are not always coordinated. We propose that particularly in cancer, conditions by which mTORC1 is activated despite a shortage in AAs can readily develop. We reason that under these conditions, GCN2 regulation becomes critical, as it operates solo, without the support of mTORC1 suppression. To explore this possibility, we generated cellular models that harbor direct mTORC1-activation mutations, such as the removal of NPRL2 and TSC2. In all cellular models, mouse and human, and regardless the type of tumor, hematopoietic and solid, ATF4 levels were strongly induced upon short AA starvation conditions (Figure 1, Figure 2 and S2). While activation of mTORC1 indirectly by insulin promoted the expression of ATF4 in an ISR independent manner (13), under acute AA starvation ATF4 expression was dependent on GCN2 (Fig. 2). Moreover, we observed that when mTORC1 activity is enforced upon AA depletion, GCN2 activity is increased in an AA-sensing independent manner (Fig. 3).

The crosstalk between GCN2 and mTORC1 activities is evolutionarily conserved. While the molecular details of the regulation vary between yeast and mammalian cells, the overall findings indicate primarily a role for GCN2 in curtailing mTORC1, either directly, as occurs in yeast (16), or indirectly, as occurs in higher eukaryotes (20). This GCN2-to-mTORC1 regulation promotes adaptation in cells (36), and in mouse models (37). In contrast, examples of direct effects of mTORC1 on GCN2 activity have not been shown. We identified a novel phosphorylation site in the charged linker domain of GCN2 (Figs. 5 and S7), which is most likely a result of a direct physical interaction between GCN2 and mTOR. The interaction is labile and highly sensitive to detergents (Figs. 4 and S6). Furthermore, only a fraction of GCN2 was phosphorylated at a steady state (Fig. 5). The profound effect that the mutations of GCN2 at S230 had on cell growth and survival following prolonged AA starvation (Fig. 6), suggests that rapid rounds of phosphorylation and dephosphorylation occur at this position. However, this dynamic has not been addressed here.

The expression of GCN2 S230D alone is not sufficient to induce the expression of ATF4 under AA starvation conditions (Figs. 5 and 6). Hence, ATF4 expression is supported by additional mTORC1 dependent activities, one may be the induction in the expression of uORF1, a prerequisite for skipping uORF2 in the mRNA of ATF4. While not affecting ATF4, the mere expression of GCN2 S230D suppressed the growth of MIA PaCa-2 cells under normal growth conditions, while providing cytoprotection under prolonged AA starvation. These opposite effects underscore the intricacy of the role of GCN2, and ATF4 in cancer cell growth. It was shown that excessive ISR can cause vulnerability in certain epithelial cancers and is not compatible with the high biosynthetic demand for rapid growth (34). However, most evidence implicate the ISR as pro-malignant by assisting cancer to survive harsh conditions. In this respect the use of the ISR inhibitor ISRIB has shown to have anti-cancer effects in breast cancer (38), prostate cancer (39), and also hematological tumors (40). This suggests that some cancers endure constant stress conditions for which the ISR provides adaptation. Since cancers often upregulate mTORC1, it suggests that the phosphorylation of GCN2 by mTORC1 may be more general. The importance of this regulation, particularly for tumors in which PI3K activity is induced, is warranted.

GCN2 operates as a dimer fostered by its autophosphorylation (41). The mechanism by which the phosphorylation of serine 230 elevates GCN2 activity can be related to improved dimerization. Alternatively, GCN2 interacts with additional regulators, besides mTORC1, such as eEF1A (42) and the ribosome P-stalk (43). The phosphorylation on S230 may affect these interactions and modify the cellular response to AA starvation. In accordance with this hypothesis, the deletion of GCN2 in cells and mice strongly promotes the activity of mTORC1, determined by 4E-BP1 and S6K1 phosphorylation (44), far more than when GCN2 is inhibited pharmacologically. This discrepancy may be due to off-target activities of the GCN2 inhibitors (24, 28), or suggest that the physical presence of GCN2 has roles beyond its catalytic activity. Based on the physical interaction of GCN2 and mTOR, we propose that additional interactions may exist, allowing GCN2 to exert non-catalytic regulatory functions. This can be addressed by comparing GCN2 KO cells to cells reconstituted with GCN2 mutants that are catalytically inactive.

## Conclusion

GCN2 activation and mTORC1 inhibition cooperate for an optimal response to the lack of AAs. However, the response of mTORC1 also to PI3K signaling which is subjected to activation irrespectively to AA availability renders mTORC1 activated. This may impose a metabolic constraint, primarily for protein synthesis. Our study describes a safety mechanism by which mTORC1, through direct phosphorylation, instructs for a higher activation of GCN2 and elevation in ATF4 expression, the main effector for adaptation. This allows cells to curtail growth and improve survival. The implication to physiology remains to be elucidated.

## Experimental procedures

### Cell culture

HEK293T, GL261, B16F10, and MIA PaCa-2 cells were cultured in DMEM (Sigma-Aldrich, D5796), RPMI8226 cells were cultured in DMEM/F-12 (Sigma-Aldrich, D6421), MM.1S cells were cultured in RPMI 1640 (Gibco, 21875034). All culture mediums were supplemented with 10% fetal bovine serum (FBS) (Gibco, 12657029), 2 mM L-glutamine (Biological Industries, 03-020), 1% penicillin-streptomycin solution (Biological Industries, 03-031), and 1 mM sodium pyruvate (Biological Industries, 03-042). Cells were maintained in humidified and sterile incubator constantly supplemented with 5% CO_2_ and maintained at 37 °C.

### Amino acid starvation assay

Cells were seeded in fresh medium at 60 to 70% confluency and incubated overnight. The following day, cells were collected, washed with phosphate-buffered saline (PBS), and cultured in either amino acid-replete or amino acid-deplete mediums as detailed in Tables S1 and S2.

### Gene editing using CRISPR-Cas9 system

Cas9 along with its single-guided RNA (sgRNA) were introduced to cells either by transfection or transduction using lentiCRISPR v2 plasmid (addgene #52961 (45)). sgRNAs targeting genes of interest were designed by using CRISPick (46, 47) and are listed below:

Human TSC2: 5′-CAGAGGGTAACGATGAACAG-3′.

Human NPRL2: 5′-GGTTGAAGAGGAGAGCATTG-3′.

Human GCN2: 5′-GAACTGGCCAAGAAACACTG-3′.

Mouse TSC2: 5′-CACAGGGTGATAATGAACAG-3′.

Cas9-sgRNA expressing cells were selected by treatment with 0.5 to 1 μg/ml puromycin (Sigma-Aldrich, P8833) for 72 h. To generate complete gene knock-out (KO), cells were further seeded at one cell per well in 96-well plate. Single-cell colonies were expanded and screened by immunoblotting of the targeted protein.

### DNA transfection

Cells were first plated in a fresh medium at a confluency of 60 to 70% for 24 h. Then, a plasmid of interest was resuspended in Opti-MEM medium (Gibco, 31985070) and incubated for 10-min then thoroughly mixed with X-tremeGENE 360 Transfection Reagent (Roche, XTG360-RO) at ratio of 1 μg to 3 μl, respectively, then incubated for 20-min. This mixture was dropwise on the plated cells which were cultured thereafter for an additional 24 to 72 h with fresh medium replacement every 24-h.

### Lentiviral transduction

HEK293T cells were transfected with the lentiviral transfer plasmid of interest along with lentiviral packaging plasmid pCMV-dR8.2 dvpr (addgene #8455) and envelope plasmid pCMV-VSV-G (addgene #8454) at ratio of 3:2:1 respectively. Lentiviral-containing medium was collected 48- and 72-h post-transfection, filtered through 0.45 μm (Cytiva, 10462100), and either used directly or frozen at −80 °C for future use. Cells to be transduced were plated to reach 50 to 60% confluency, then lentiviral containing medium was added along with Polybrene (Millipore, TR-1003-G) at final concentrations of 33.34% and 10 μg/ml, respectively. Transduced cells were selected after 24-h by a 72-h treatment with 0.5 to 1 μg/ml puromycin.

### Immunoblotting

Cells were collected, washed with PBS, and centrifuged at 4000*g* and 4 °C for 3 min. Cell pellets were either frozen at −80 °C for future lysis or lysed directly using ice-cold lysis buffer (50 mM Tris-HCl pH = 7.5, 150 mM NaCl, 0.2% CHAPS, 0.1% SDS, 1% IGEPAL CA-630) supplemented with proteases and phosphatases inhibitors (TargetMOI, C0001, C0002, and C0003). Lysates were incubated on ice for 10 min, then centrifuged at 19,000*g* and 4 °C for 15 min. Produced supernatants were then separated, quantified, and mixed with sample buffer (180 mM Tris-HCl pH = 6.8, 7% SDS, 30% glycerol, 400 mM DTT, 0.003% bromophenol blue) at ratio of 3:1, respectively. Samples were denatured by heat (95 °C for 5 min) and then loaded onto SDS-polyacrylamide gel (SDS-PAGE) fixed within 1.5 mM spaced glasses and submerged in running buffer (25 mM Tris, 192 mM glycine, 0.1% SDS), and then resolved by electrophoresis (120–150 V for 1–1.5-h). SDS-PAGE was then transferred onto PVDF/nitrocellulose membrane by electrophoresis (100 V for 1.5 h) in transfer buffer (25 mM Tris, 192 mM glycine, 10% methanol). Blotted membrane was blocked with 5% skim milk (BD Life Sciences, 232100) dissolved in TBST buffer (10 mM Tris-HCl pH = 8, 150 mM NaCl, 0.1% Tween) for 1 h at RT and then washed 3 times with TBST. Blocked membrane was incubated with primary antibody solution (0.01% antibody in 5 ml TBST and 0.1% NaN_3_) in rolling tube for overnight at 4 °C. Membrane was then washed 3 times with TBST and incubated with Horseradish peroxidase (HRP) conjugated secondary antibody for 1 h at RT, washed again and then detected by chemiluminescence using Bio-Rad ChemiDoc XR. Immobilon Crescendo (Millipore, WBLUR0500) was used as chemiluminescent substrate. Antibodies that were used are listed here: anti-ATF4 (CST #11815), anti-p97 (was kindly provided by Dr Ariel Stanhill, (The Open University, Israel), anti-P-eIF2α-S51 (CST #3398), anti-eIF2α (CST #2103), anti-P-S6K1-T389 (CST #9205), anti-S6K1 (CST #9202), anti-GCN2 (CST #65981), anti-GCN2 (CST #3302), anti-P-GCN2-T899 (abcam, ab75836), anti-B-actin (CST #8457), anti-mTOR (Millipore, 04-385), anti-Raptor (CST #2280) anti-P-S6-S240/244 (CST #5364) anti-S6 (CST #2217), anti-FLAG (Sigma-Aldrich, F1804), anti-TSC2 (CST #4308), anti-NPRL2 (CST #37344), anti-P-4EBP1-T37/46 (CST #9459), anti-4EBP1 (CST #9644), goat anti-mouse (Jackson ImmunoResearch, 115-035-003), and goat anti-rabbit (Jackson ImmunoResearch, 111-035-003).

### Immunoprecipitation

Cell pellets were first resuspended in ice-cold, detergent-free lysis buffer (50 mM Tris-HCl pH = 7.5, 500 mM NaCl) supplemented with protease inhibitors, and then homogenized by using Dounce homogenizer. Fifty strokes were applied to each sample followed by incubation on ice for 10 min. Homogenates were centrifuged twice at 19,000*g* and 4 °C for 15 min. Produced supernatants were quantified and then mixed with pre-washed anti-FLAG magnetic beads (Sigma-Aldrich, M8823) at ratio of 1 mg of total protein to 10 μl beads, respectively, in total volume of 1 ml. Samples were incubated in rolling Dolphin microcentrifuge tubes for overnight at 4 °C. Then beads were washed for 5 times with washing buffer (50 mM Tris-HCl pH = 7.5, 150 mM NaCl, 0.001% IGEPAL CA-630) and finally resuspended in sample buffer. Samples were denatured by heat (95 °C for 5 min) and then loaded onto SDS-PAGE.

### Plasmid cloning

Human GCN2 coding sequence (NCBI Reference Sequence: NM_001013703.4) was flanked with NotI and PacI restriction sites, synthesized, and cloned into pUC57 plasmid by Syntezza Bioscience Ltd. Flanking sequences are: 5′-AGCTTAATTAAGTCCAAC and GGAGGCAGCGCGGCCGCGGCGA-3′. This plasmid was restricted with PacI (NEB, R0547S) and NotI (NEB, R3189L) and GCN2 was purified and cloned in-frame with 3xFLAG into pLJC2 (Addgene, #87975) vector backbone. To generate CRISPR/Cas9-insensitive GCN2 expression plasmid, 6 nucleotides in the used sgRNA scaffold were mutated using Q5 Site-Directed Mutagenesis Kit (NEB, E0554S). The following primers were used:

Forward: 5′-AAAAGCATTGTGGGGAGGTGATGATCTTTG-3′.

Reverse: 5′-TAGCTAGCTCTTCTAGGCGAGATTTTAACAAATTG-3′.

To clone truncated GCN2 into pLJC2 vector, primers were designed to amplify the domain of interest using the CRISPR/Cas9-insensitive vector as a template and Q5 Hot Start High-Fidelity DNA Polymerase (NEB, M0493S), primers were as follows:

20 to 1016 domain:

Forward: 5′-AGCTTAATTAAGTCCAACATGCCGCAACGACAGGACCACGA-3′.

Reverse: 5′-TCGCCGCGGCCGCGCTGCCTCCGTGCAGCACTTCATGCAGCT-3′.

1 to 276 domain without FLAG:

Forward: 5′-AGCTTAATTAAGTCCAACATGGCTGGGGGCCGTGGGGC-3′.

Reverse: 5′-CGCGGCCGCTTAATCAGGACTCCCCATATTGA-3′.

1 to 276 domain with FLAG:

Forward: 5′-AGCTTAATTAAGTCCAACATGGCTGGGGGCCGTGGGGC-3′.

Reverse: 5′-CGCGGCCGCATCAGGACTCCCCATATTGA-3’.

The following primers were used to generate site-specific mutations using Q5 Site-Directed Mutagenesis Kit:

L595A + K610A.

Forward: 5′-GCTGTCATCAAGGTGCAGAACGCGTTGGACGGCTGCTGCTAC-3′.

Reverse: 5′-TCCAAAAGCTCCTTTACCAAGAGCTTGTAATTCTTCAAACTCAATGAAGTATCGG-3′.

P1003A + Q1005A.

Forward: 5′-CCCGCGATGGAGGAGTCAGAGCTGCATGAAGTGCTG-3′.

Reverse: 5′-TGGAGCCAGCAGCTCACTCTTGAGCAGTTCTGTGGCTGTGGG-3′.

S230A.

Forward: 5′-ACATGGAGGCGCTCCTGACTTTGTAGGAAATGGTAA-3′

Reverse: 5′-AGAATGGCAGCCGTTCTGTGTCCTCCTGGGTCCTTC-3′

S230D.

Forward: 5′-ACATGGAGGCGATCCTGACTTTGTAGGAAATGGTAA-3′

Reverse: 5′-AGAATGGCAGCCGTTCTGTGTCCTCCTGGGTCCTTC-3′

All generated plasmids were sequenced to confirm mutations were correctly achieved.

### Cells counting

Cells were counted using flow cytometer (Beckman Coulter, CytoFlex). Cell samples were collected in equal volumes and stained with propidium iodide (PI) (Sigma-Aldrich, P4170) at a final concentration of 1 μg/ml. Samples underwent analysis using CytExpert software with the following settings: gain channels of forward and side scatter were set both on 12 and PE channel on 1. The volume recorded for each sample was set to 10 μl. Cell debris and PI-stained cells were subtracted from the total event counts equally across all samples.

### *In vitro* kinase assay

FLAG-mTOR, FLAG-Rheb (addgene, #165031) and FLAG-1-276-GCN2 were independently expressed, immunoprecipitated and purified from GCN2-TSC2 DKO HEK293T cells. Immunoprecipitation was done as previously described but with the addition of CHAPS (0.05%) to the lysis buffer. Immunoprecipitated FLAG-1-276-GCN2 was subjected to dephosphorylation with Calf Intestinal Alkaline Phosphatase (NEB, #M0290) at RT for 30 min. Beads were washed thoroughly for 5 times and then proteins were eluted by agitation with 2.5 mM 3xFLAG peptide (MCE, HY-P0319) at 4 °C for 30 min. To perform the kinase assay, purified proteins were mixed in kinase buffer (25 mM Tris-HCl pH = 7.5, 5 mM beta-glycerophosphate, 2 mM DTT, 0.1 mM Na3VO4, 10 mM MgCl_2_) supplemented with 200 μM ATP and incubated at 30 °C for 30 min. Sample buffer was added subsequently to terminate the reaction and analyze the phosphorylation using phospho-tag SDS-PAGE.

## *In silico* docking and molecular dynamic simulations

### Protein preparation

The crystal structures of human mTOR kinase (chain A in pdb-id: 4JSV, 3.5 Å) (48) and GCN2 kinase (chain A in pdb-id: 7QWK, 2.3 Å) (49) were retrieved from the Protein Data Bank and prepared using default settings in the protein preparation protocol in the Molecular Operating Environment (MOE) [3]. This includes the incorporation of hydrogen atoms, modeling of possible missing loops, and optimization of the hydrogen bonding network by adjusting protonation and tautomeric states of relevant residues at pH = 7. The prepared structures were subjected to geometry refinement using the Amber10ff.

### Protein–protein docking

The protein–protein docking meta-approach (50) was employed to integrate the benefits of 7 docking engines: PatchDock (51), HDOCK (52, 53), PyDockWeb (54), FireDock (55), LZerD (56), ClusPro (57), and MOE [3], to generate a “consensus-based” prediction of the mTOR-GCN2 complex. The docking protocol starts with performing a series of blind protein–protein docking calculations using the protein docking engines above. The top ten predicted complexes from each docking engine were chosen for the conformational clustering step. The total 70 predicted complexes were clustered based on the RMSD values of all heavy atoms using the Schrödinger program package (Schrödinger v2022-1: Maestro, Schrödinger, LLC, 2020). The optimum number of clusters, was determined from Kelley penalty plots (58). Finally, the model nearest to the centroid of the most populated cluster was considered as the final model.

### MD simulation

The consensus model from protein-protein docking was subjected to a 100 ns long restrained molecular dynamics (MD) simulation in order to further refine the predicted mTOR-GCN2 complex by extending the conformational sampling and studying the dynamics and stability of the complex over time. The MD simulation was conducted in the NPT ensemble using the Desmond engine (59) with the OPLS4 force field (60) and multi-time-step RESPA integrator (61) with bonded, near-nonbonded (short-range van der Waals forces) and far-nonbonded (long-range electrostatic forces) timesteps of 2.0, 2.0, and 6.0 fs, respectively. The mTOR-GCN2 complex was explicitly solvated in a cubic simulation box with a 10 Å buffer in each direction using the TIP3P water model (62). The periodic boundary conditions were applied in all directions in the MD simulations. The system was neutralized by incorporating an appropriate number of Na^+^/Cl^−^ ions, and a NaCl salt concentration of 0.15 M was considered. The Nosé–Hoover thermostat (63) (relaxation time of 1 ps) and Martyna–Tobias–Klein barostat (64) (relaxation time of 2 ps) were utilized to control the temperature and pressure of the system at 300 K and 1 atm, respectively. The Long-range electrostatic forces were calculated using the particle-mesh Ewald method (65, 66). The SHAKE algorithm (67) was applied to the covalent bonds between all heavy atoms and hydrogens with maximum iterations and tolerance of 8 and 10^−8^, respectively. The standard minimization and equilibration protocol was used as follows: (i) NVT Brownian dynamics with restraints on solute heavy atoms at T = 10 K for 100 ps, (ii) NVT simulation with small timestep at T = 10 K with restraints on solute heavy atoms for 12 ps, (iii) NPT MD simulation at T = 10 K with restraints on solute heavy atoms for 12 ps, (iv) NPT MD simulation at T = 300 K with restraints on solute heavy atoms for 12 ps, and (v) NPT MD simulation at T = 300 K without restraints for 24 ps.

### Free energy of binding and alanine scanning

A series of 100 snapshots from the trajectory of MD simulation (*i.e.*, every 1 ns) were recorded to calculate the free energy of binding between the mTOR and GCN2 proteins. The Schrödinger package v.2021-1 was employed to calculate the free energy of binding using the Molecular Mechanics Generalized Born Surface Area (MM-GBSA) technique (68). The key residues that play a pivotal role in the formation of the mTOR-GCN2 complex were identified by assessing alterations in protein binding affinity (ΔAffinity) resulting from mutations of all residues in the interface of 2 protomers (5 Å distance) to alanine. This evaluation was conducted using BioLuminate alanine scanning calculations (69), a method available in the Schrodinger package. The change in binding affinity is calculated from a thermodynamic cycle described in the reference (50). For each mutation, the free energy calculation was conducted using the MM-GBSA method which incorporates an implicit solvation model. A positive value of ΔAffinity signifies that the muted protein exhibits weaker binding compared to their parent counterparts.

### Sample preparation for MS analysis

Packed beads were resuspended in 100 μl 8M urea, 10 mM DTT, 25 mM Tris-HCl pH 8.0 and incubated for 30 min at room temperature. Iodoacetamide was added to a final concentration of 55 mM followed by incubation of 30 min in the dark. Eight volumes of 25 mM Tris-HCl pH 8.0 were added to dilute the urea and 0.4 μg/sample of Trypsin was added and incubated overnight at 37 °C with gentle agitation. Next day the peptides were acidified using formic acid to final concentration of 0.38% and desalted on home-made C18 Stage tips.

### nanoLC-MS/MS analysis

MS analysis was performed using a Q Exactive Plus mass spectrometer (Thermo Fisher Scientific) coupled online to a nanoflow UHPLC instrument, Ultimate 3000 Dionex (Thermo Fisher Scientific). Peptides (0.45 μg, as estimated by O.D. 280 nm) were separated over a non-linear gradient (0–80% acetonitrile) run at a flow rate of 0.15 to 0.3 μl/min on a reverse phase 25-cm-long C18 column (75 μm ID, 2 μm, 100 Å, Thermo PepMapRSLC) for 120 min. The survey scans (380–2000 m/z, target value 3E6 charges, maximum ion injection times 50 ms) were acquired and followed by higher energy collisional dissociation (HCD) based fragmentation (normalized collision energy 25). A resolution of 70,000 was used for survey scans and up to 15 dynamically chosen most abundant precursor ions, with “peptide preferred” profile were fragmented (isolation window 1.6 m/z). The MS/MS scans were acquired at a resolution of 17,500 (target value 1E5 charges, maximum ion injection times 120 ms). Dynamic exclusion was 60 s. Data were acquired using Xcalibur software (Thermo Scientific). To avoid a carryover, the column was washed with 80% acetonitrile, 0.1% formic acid for 25 min between samples.

### MS data analysis

Mass spectra data were processed using the MaxQuant computational platform. Peak lists were searched against the human reference proteome (UP000005640) obtained from UniProt. The search included cysteine carbamidomethylation as a fixed modification and oxidation of methionine as variable modifications and allowed up to 2 miscleavages. The match-between-runs option was used. Required FDR was set to 1% at the peptide and protein level. Relative protein quantification in MaxQuant was performed using the label-free quantification (LFQ) algorithm.

## Data availability

Data underlying this article are available in the article and in its Supplementary material. Original mass spectrometry data will be provided upon request.

## Supporting information

This article contains supporting information.

## Conflict of interest

The authors declare that they have no conflicts of interest with the contents of this article.
