# Supplementary material for: Phosphorylation of GCN2 by mTOR confers adaptation to conditions of hyper-mTOR activation under stress
Source: J Biol Chem. 2024 Jul 14;300(8):107575. doi: 10.1016/j.jbc.2024.107575 (PMC11362803; doi:10.1016/j.jbc.2024.107575)
Supplement: Supporting information [file mmc1.pdf]

Table 1S:

Components	AA-replete medium (1ml)	AA-deplete medium (1ml)
L-Glutamine 200 mM (Biological Industries, 03–020)	10 µl	0
Essential amino acids (ThermoFisher Scientific, 11130036)	20 µl	0
Non-essential amino acids (Biological Industries, 01-340-1B)	10 µl	0
Distilled water	0	40 µl
D-Glucose 1110 mM (Gibco, A2494001)	22.5 µl	22.5 µl
Dialyzed FBS (Biological Industries, 04-011-1A)	100 µl	100 µl
DMEM/F-12 (USBiological, D9807–11)	837.5 µl	837.5 µl

Table 2S:

Composition of the solution of Essential amino acids:

Components	mM
L-Arginine hydrochloride	30
L-Cystine	5
L-Histidine hydrochloride-H2O	10
L-Isoleucine	20
L-Leucine	20
L-Lysine hydrochloride	20
L-Methionine	5
L-Phenylalanine	10
L-Threonine	20
L-Tryptophan	2.5

L-Tyrosine	10
L-Valine	20

The non-essential amino acids solution contains 10 mM each of the following amino acids: L-Alanine, L-Asparagine·H_2O, L-Aspartic acid, L-Glutamic acid, Glycine, L-Proline, L-Serine.

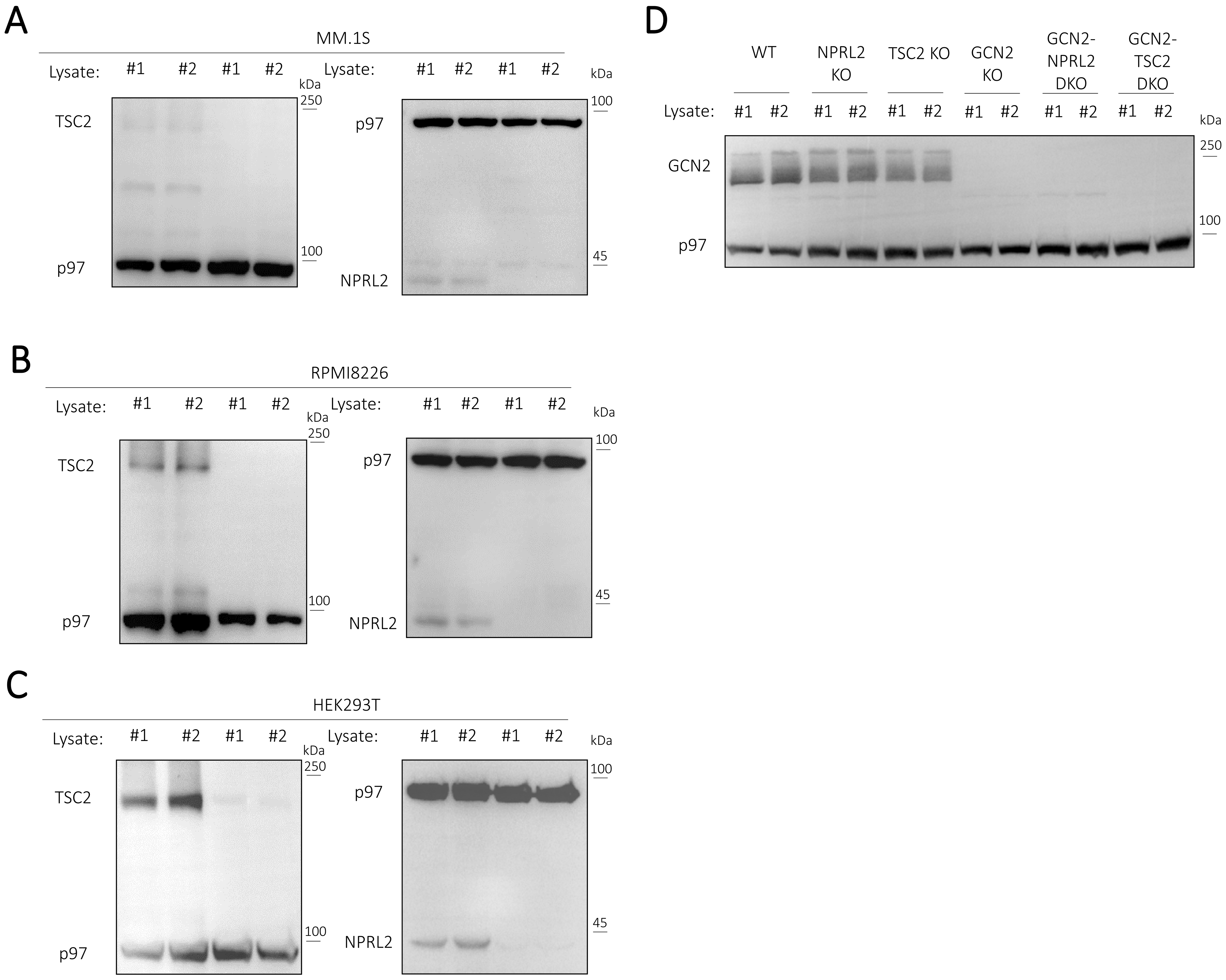

Figure S1: CRISPR/Cas9-mediated gene deletion of TSC2, NPRL2 and GCN2. Generation of TSC2 and NPRL2 KO cells in MM.1S (A), RPMI8226 (B) and HEK293T (C) and/or GCN2 KO in HEK293T (D). Shown are typical immunoblots out of two independent cell lysates of TSC2 and NPRL2 with their p97 as a loading control.

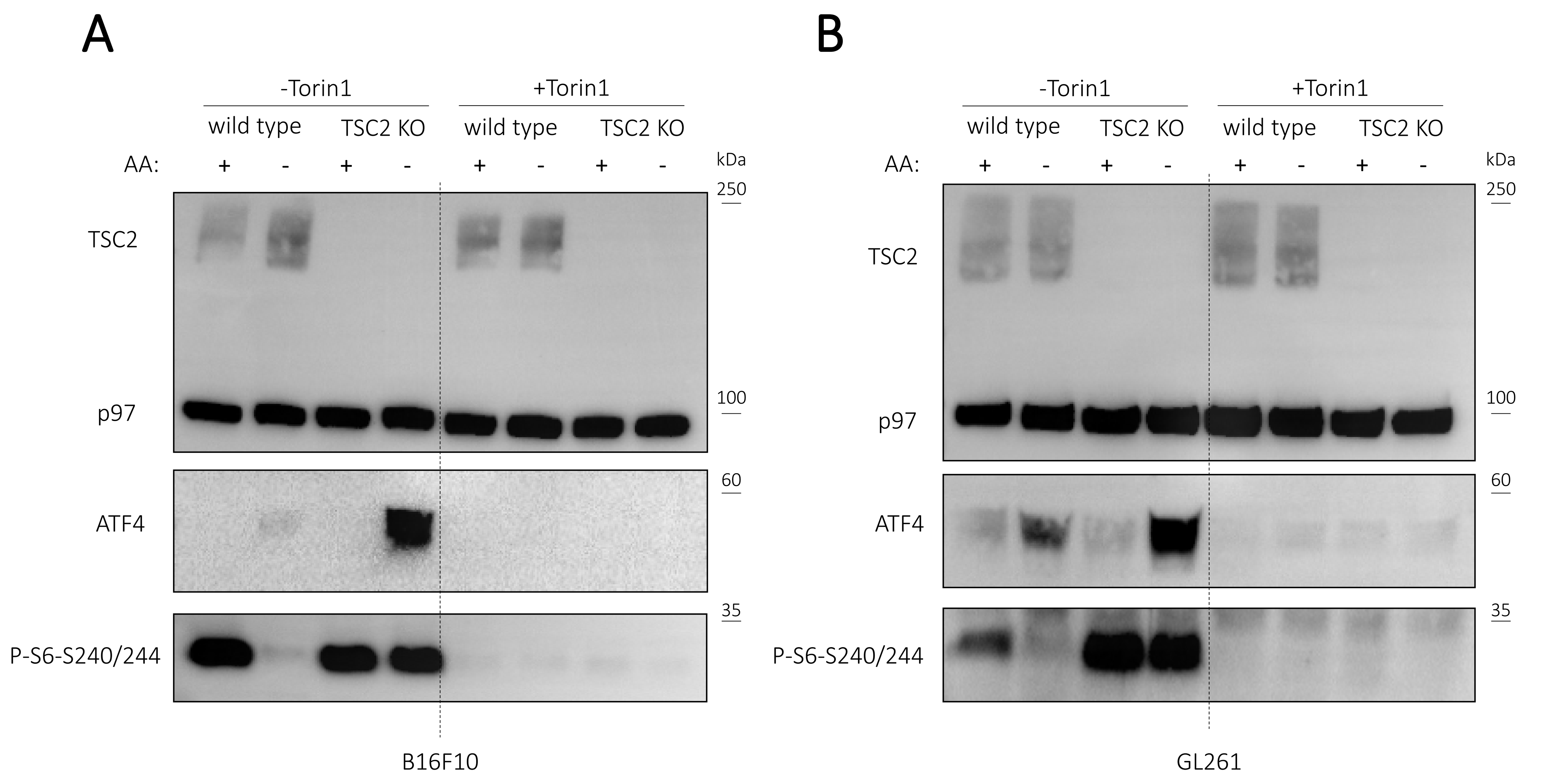

Fig. S2: mTORC1-induced ISR activation is conserved in mouse cell lines. B16F10 (A) or GL261 (B) wild type and TSC2 KO cells were cultured in AA replete, or AA deplete conditions for 1h and either treated with Torin1 [1 µM] or left untreated as a control.

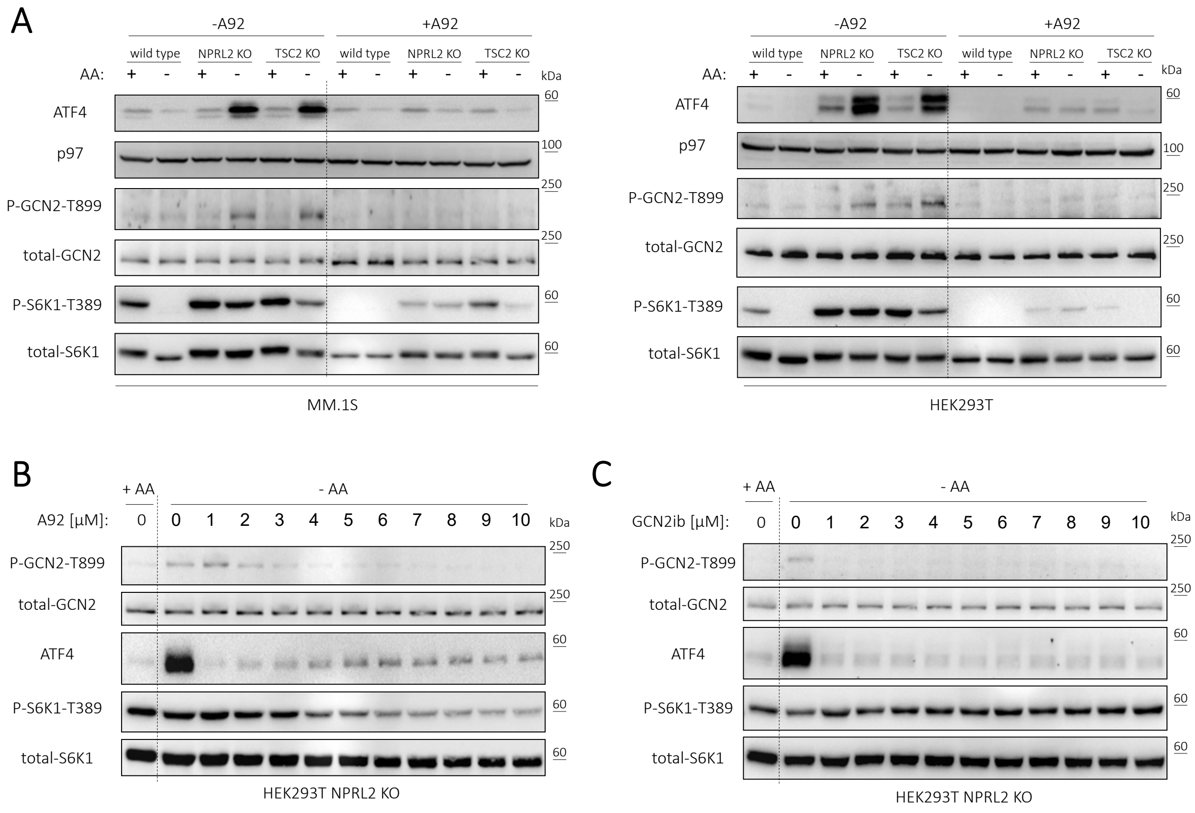

Fig. S3: The effect of GCN2 inhibitors on mTORC1-mediated activation of the ISR. (A) Wild type, NPRL2 KO, and TSC2 KO cells from the indicated cell lines were cultured in either AA replete or AA deplete conditions. Cells were treated with A92 (10 µM) or left untreated as a control. (B) Dose-response analysis of A92 on HEK293T NPRL2 KO cells cultured in AA deplete condition for 1h. (C) Dose-response analysis of GCN2iB on HEK293T NPRL2 KO cells cultured in AA deplete condition for 1h.

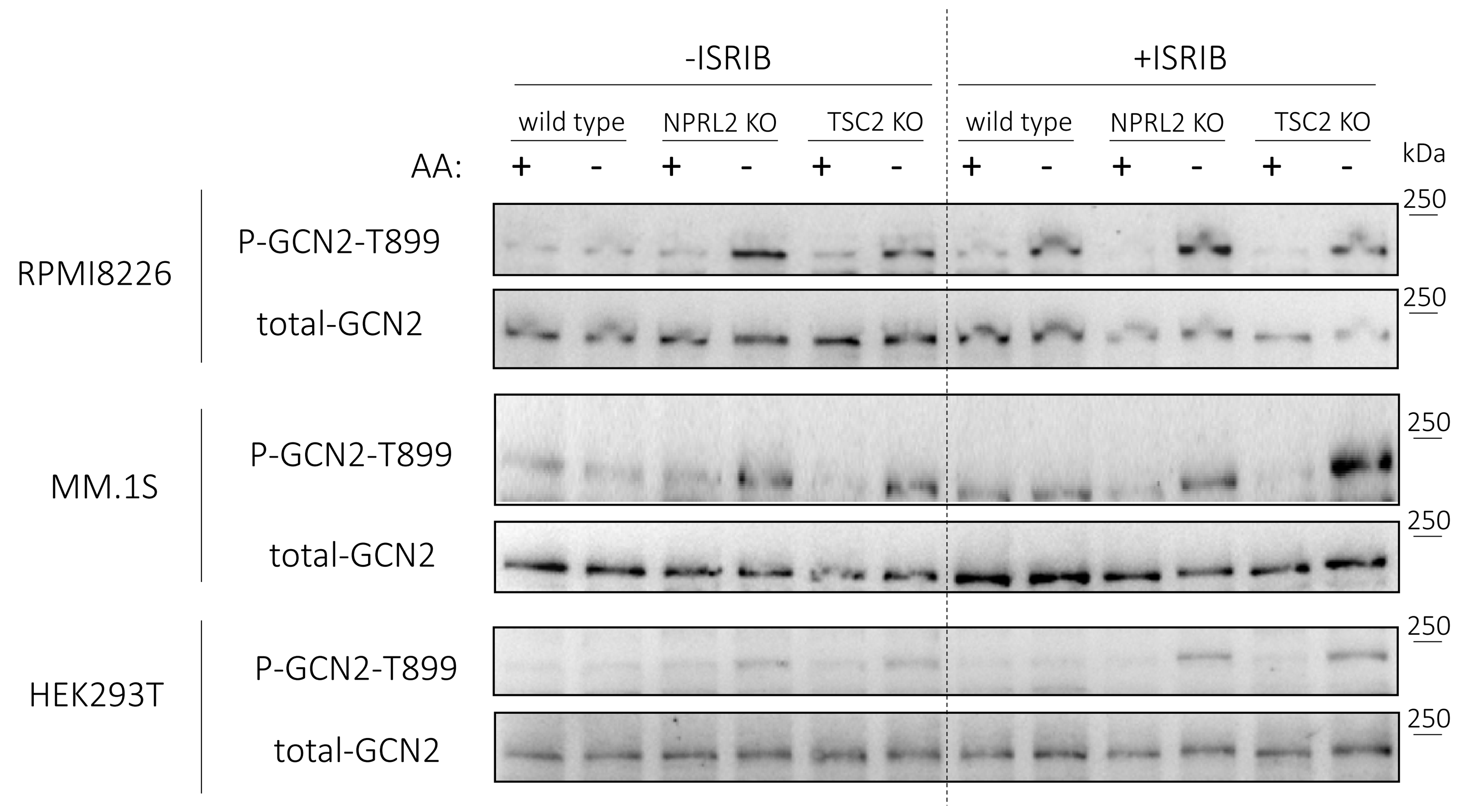

Fig. S4: The effect of ISRIB on GCN2 activity. Wild-type, NPRL2 KO, and TSC2 KO cells of the indicated cell lines were cultured in AA replete, or AA deplete conditions and either treated with ISRIB [1 µM] or left untreated as a control.

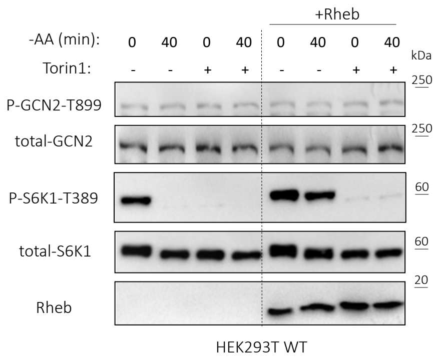

Fig. S5: The effect of RHEB overexpression on endogenous GCN2 activity. HEK293T wild type cells were transfected with RHEB, and then subjected to AA starvation for the indicated times, and either treated with Torin1 [1 μM] or left untreated as a control. GCN2 autophosphorylation was assessed by immunoblotting against P-GCN2-T899 and total-GCN2. mTORC1 activity was assessed by immunoblotting against P-S6K1-T389/total-S6K1.

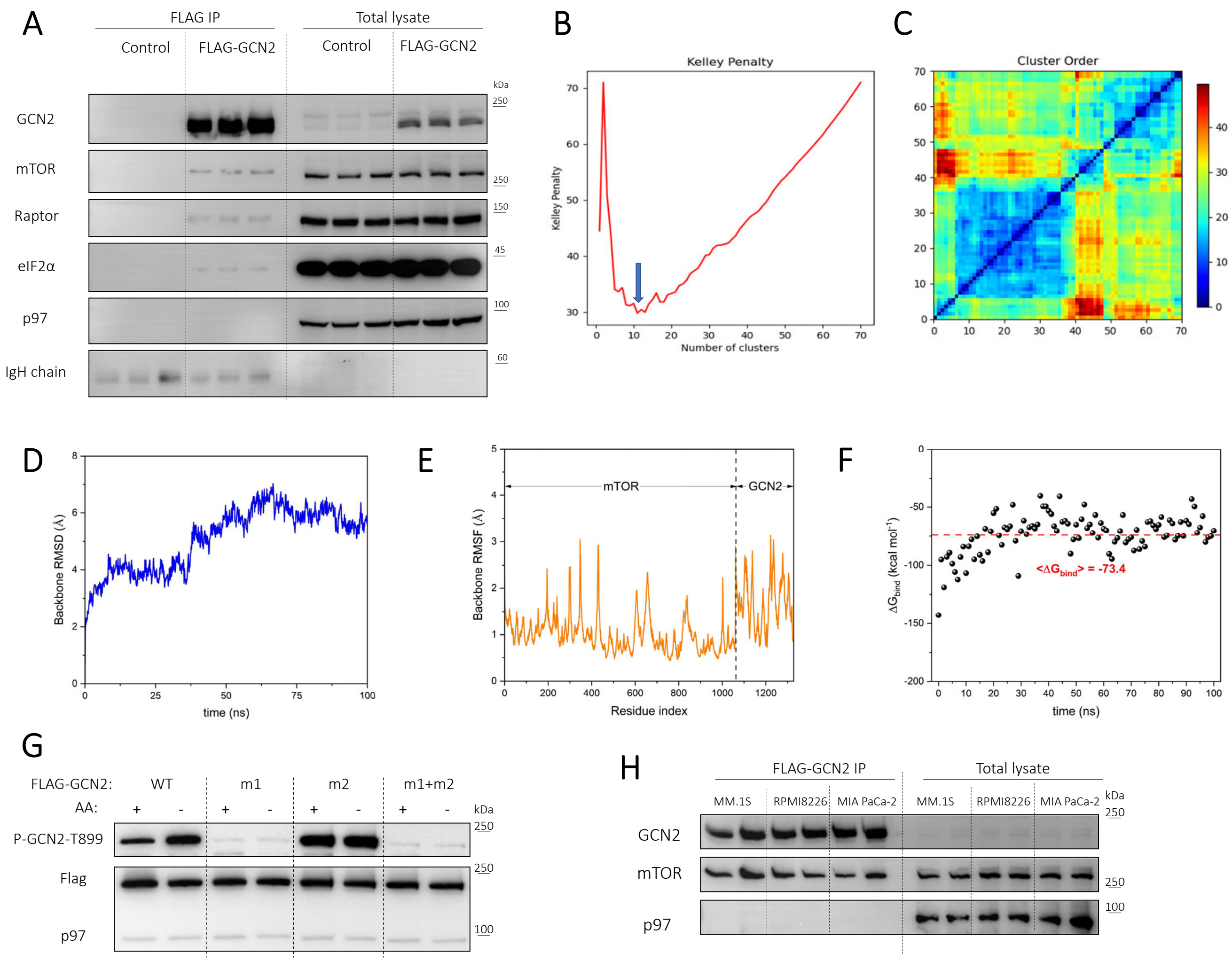

Figure S6: In vitro and in silico interaction of mTOR and GCN2. (A) Protein complexes were extracted in detergent-free lysis buffer following transfection of empty vector or FLAG-GCN2 into HEK293T cells. Lysates were subjected to immunoprecipitation using flag beads. Shown are representative immunoblots of both flag-immunoprecipitated proteins (n=3 for each condition, technical replicates) and their total lysates (n=3, technical replicates). (B) Kelley penalty plot and (C) distance matrix obtained from protein-protein docking meta-approach for mTOR/GCN2 conformation clustering. The optimum number of clusters (11) is pointed by a blue arrow. (D) Backbone RMSD, (E) backbone RMSF, and (F) free energy of binding (ΔG_{bind}, MM-GBSA technique) plots for mTOR/GCN2 protein complex during 100 ns MD simulation. (G) HEK293T GCN2 KO cells were transfected with the indicated FLAG-GCN2 and subjected to AA starvation for 2h. Shown are typical immunoblots of the indicated proteins. (H) Immunoprecipitation of FLAG-GCN2 from the indicated cell lines was done as described in A. Shown are representative immunoblots of both flag-immunoprecipitated proteins (n=2, technical replicates) and their total lysates (n=2, technical replicates).

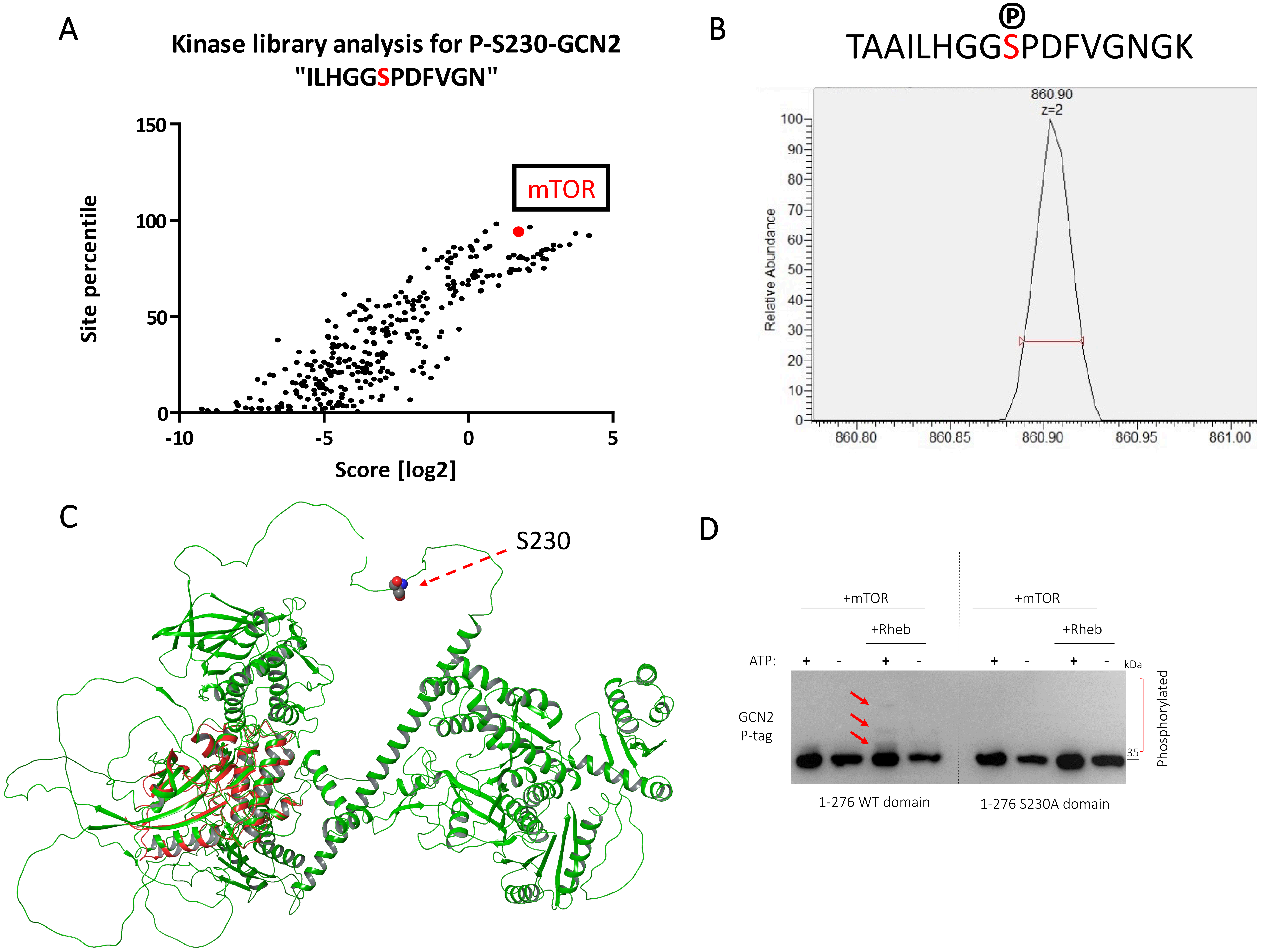

Figure S7: S230 is an mTOR sensitive phosphorylation site. (A) Kinase prediction for S230-GCN2 motif using Kinase Library atlas (PMID: 36631611) scored mTOR as highly potential kinase. (B) Shown the LC-MS chromatogram of the phosphorylated peptide P-S230. (C) Shown is the AlphaFold model of the human full-length GCN2 protein, with its S230 phosphorylation site annotated. In red is the region of GCN2 that was crystalized and used for docking. (D) Shown is a representative phospho-tag SDS PAGE for WT or S230A 1-276 domains that were incubated with immunoprecipitated mTOR from cells that either overexpress or not Rheb. Potential mTOR-mediated phosphorylation of GCN2 are indicated by arrows.

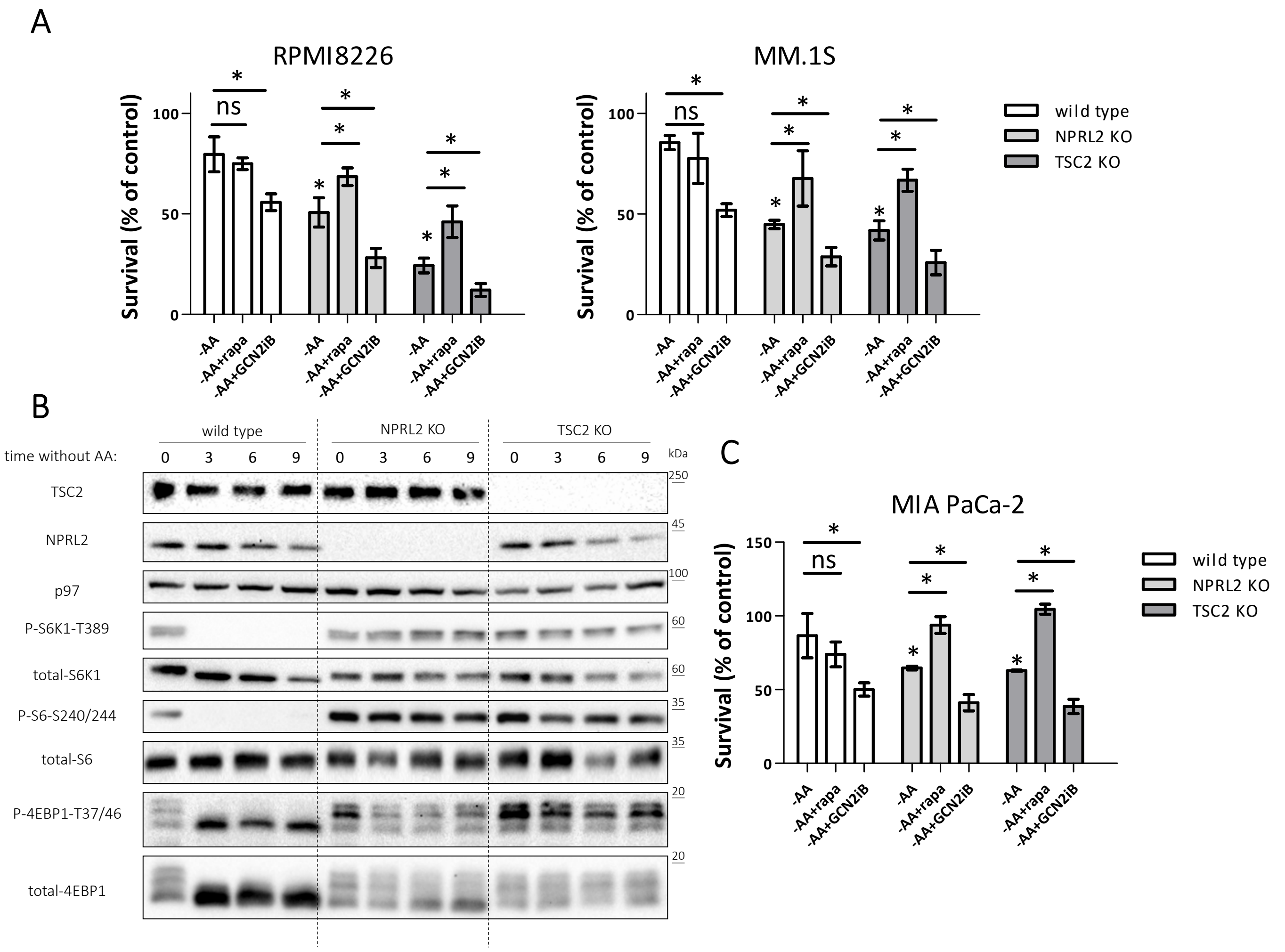

Figure S8: mTORC1 activation compromises cell viability under AA starvation conditions. (A) Column graph represents cells viability following AA starvation and treatment with rapamycin [50 nM] or GCN2iB [1 µM] for 6h for RPMI8226 cells and for 12h for MM.1S cells. Shown is average relative viability of three independent experiment±SD, *p<0.05 of unpaired two-tailed student's t-test, ns, not significant. (B) Generation of NPRL2 KO and TSC2 KO in MIA PaCa-2 cells. Cells were starved from AA for the indicated time points and mTORC1 activity was assessed by immunoblotting of its downstream targets. Shown are representative immunoblots for the indicated proteins. (C) Column graph represents cells viability of MIA PaCa-2 cells following AA starvation and treatment with rapamycin [50 nM] or GCN2iB [1 µM]. Shown is average relative viability of three independent experiment±SD, *p<0.05 of unpaired two-tailed student's t-test, ns, not significant.
